# A combination of two ELISA tests for nasopharyngeal carcinoma screening in endemic areas based on a case-control study

**DOI:** 10.7717/peerj.10254

**Published:** 2020-11-12

**Authors:** Dongping Rao, Meiqin Fu, Yingjie Chen, Qing Liu, Lin Xiao, Xin Zhang, Zhongxiao Li, Haitao Li, Yongyi He, Yongxing Chen, Jieying Chen, Jin Hu, Yanming Huang

**Affiliations:** 1 Department of Medical Records, Jiangmen Central Hospital, Affiliated Jiangmen Hospital of Sun Yat-sen University, Jiangmen, Guangdong, China; 2 Clinical Laboratory, Jiangmen Central Hospital, Affiliated Jiangmen Hospital of Sun Yat-sen University, Jiangmen, Guangdong, China; 3 Department of Preventive Medicine, Sun Yat-sen University Cancer Center, Guangzhou, Guangdong, China; 4 Department of Radiotherapy, Jiangmen Central Hospital, Affiliated Jiangmen Hospital of Sun Yat-sen University, Jiangmen, Guangdong, China; 5 Clinical Experimental Center, Jiangmen Key Laboratory of Clinical Biobanks and Translational Research, Jiangmen Central Hospital, Affiliated Jiangmen Hospital of Sun Yat-sen University, Jiangmen, Guangdong, China; 6 Department of Ear-Nose-Throat, Jiangmen Central Hospital, Affiliated Jiangmen Hospital of Sun Yat-sen University, Jiangmen, Guangdong, China; 7 Department of Respiratory Medicine, Clinical Experimental Center, Jiangmen Key Laboratory of Clinical Biobanks and Translational Research, Jiangmen Central Hospital, Affiliated Jiangmen Hospital of Sun Yat-sen University, Jiangmen, Guangdong, China

**Keywords:** Nasopharyngeal carcinoma, Screening, Marker, Epstein-Barr virus, combination

## Abstract

For populations with a high risk of nasopharyngeal carcinoma (NPC) in Guangdong province in southern China, mass screening is the first choice to prevent death from NPC. To improve the performance of NPC screening, we used a combination based on the IgA antibody against the Epstein-Barr virus (EBV) capsid antigen (VCA-IgA) and the IgA antibody against Epstein-Barr virus nuclear antigen 1 (EBNA1-IgA) to NPC screening by enzyme-linked immunosorbent assay (ELISA). A multiplication model was applied to measure the level of the combination. We evaluated the NPC screening effect of the markers.A case-control study was performed to assess the NPC screening effect of the markers. A total of 10,894 serum specimens were collected, including 554 samples from NPC patients and 10,340 samples from healthy controls. In the training stage, 640 subjects were randomly selected, including 320 NPC cases and 320 healthy controls. In the verification stage, 10,254 subjects were used to verify the NPC screening effect of the combination. Receiver operating characteristic (ROC) analysis was performed. In the verification stage, the combination achieved an sensitivity of 91.45%, a specificity of 93.45%, and an area under the ROC curve (AUC) of 0.978 (95% CI [0.968–0.987]). Compared with VCA-IgA and EBNA1-IgA individually, the combination had an improved screening performance. A probability (PROB) calculated by logistic regression model based on VCA-IgA and EBNA1-IgA was applied to NPC screening by ELISA in China. The AUC of the combination was a little bit larger than the PROB. There was a slight increase (3.13%) in the sensitivity of the combination compared to the sensitivity of the PROB, while the specificity was lower for the combination (92.50%) than for the PROB (95.94%). We successfully applied a combination of two ELISA tests based on VCA-IgA and EBNA1-IgA for NPC screening by using a multiplication model. The results suggested that the combination was effective and can be an option for NPC screening.

## Introduction

In southern China and southeast Asia, nasopharyngeal carcinoma (NPC) is a common malignant tumour, with an incidence rate of 10–40/100,000 per year (Ng et al., 2005; Torre et al., 2015; Wei & Sham, 2005; Yang et al., 2005; Yu & Yuan, 2002). Jiangmen city, an endemic area of NPC located along the Zhujiang River in the central southern area of Guangdong province, has a high-risk NPC population. The NPC incidence rate in the Jiangmen urban area is 14.99/10^5^ (Wei et al., 2017). The population-based cancer registry was established in Jiangmen to report the incidence and mortality of cancers. Population-based NPC screening was performed in the Jiangmen urban area by Jiangmen Central Hospital from June 2018 to March 2020.

The occurrence of NPC is strongly associated with Epstein-Barr virus (EBV) infection (Fachiroh et al., 2004; Gulley, 2001; Henle & Henle, 1976; Sam, Abu-Samah & Prasad, 1994). Furthermore, host genetics, smoking, the consumption of salted fish and occupational exposures are contributors to the pathogenesis of NPC (Chang & Adami, 2006; Chang et al., 2017; Chen et al., 2019; Yong et al., 2017). The development mechanisms of NPC are unclear. Mass screening is the main effective measure to detect NPC early in endemic areas.

EBV antibodies are widely used as markers in NPC screening (Chien et al., 2001; Ji et al., 2019; Ji et al., 2007; Ng et al., 2005; Tan et al., 2020; Zeng et al., 1982). A number of studies have shown that screening for NPC by using EBV antibodies is an effective measure to improve the survival rate of NPC patients (Choi et al., 2011; Ji et al., 2007; Jia et al., 2006; Ng et al., 2010). The combined serological test based on the IgA antibody against the EBV capsid antigen (VCA-IgA) and the IgA antibody against EBV nuclear antigen 1 (EBNA1-IgA) by enzyme-linked immunosorbent assay (ELISA) was used for NPC screening in endemic areas in China (Gao et al., 2017; Liu et al., 2012; Yu et al., 2018). In previous studies, the probability (PROB) calculated by logistic regression based on VCA-IgA and EBNA1-IgA was applied to NPC screening in China (Gao et al., 2017; Liu et al., 2012; Yu et al., 2018).

Multiplication model was applied to make new maker to improve diagnostic effect (Enyedi et al., 2020). In this study, a combination of two ELISA tests based on VCA-IgA and EBNA1-IgA was applied to improve the effect of NPC screening by using a multiplication model and the NPC screening effect of the markers was evaluated.

## Materials & Methods

### Study population

A case-control study was performed to compare the effect of the NPC screening of markers, including 554 NPC cases and 10,340 healthy controls. This study included the training stage and the verification stage. The inclusion criteria for NPC cases included being histologically confirmed by biopsy, aged between 30 and 69 years, and residing in Jiangmen. A total of 554 serum specimens were continuously collected from NPC patients at Jiangmen Central Hospital from June 2018 to March 2020. Among the 554 cases, 7 (1.26%) participated in the NPC screening program. NPC stages were classified according to the 2008 staging system of China (Lin et al., 2009). The stages were divided into early-stage (stage I and stage II) and advanced-stage (stage III and stage IV) disease. A total of 554 cases comprised 73 early-stage cases and 481 advanced-stage cases. A total of 320 NPC training samples were randomly selected from the 554 NPC cases, and the remaining 234 of 554 NPC cases were used as validation samples.

A total of 10,340 healthy controls were obtained from an NPC screening programme performed in a population aged 30–69 years in the Jiangmen City urban area from June 2018 to March 2020. The healthy controls resided in Jiangmen of Guangdong province. A total of 320 training samples were randomly selected from the 10,340 healthy controls and were frequency matched to the 320 training NPC cases by age (5-year age groups) and sex. The remaining 10.020 of 10.340 healthy controls were used as the validation samples.

The information on age, sex, smoking history and family history of NPC for the cases and healthy controls were collected by inquiring medical records and using a questionnaire survey.

### Serological test

In total, 10,894 serum samples were collected and underwent serological tests in separate batches at Jiangmen Central Hospital. The samples were separated and stored at −40 °C. In this study, the NPC screening markers included VCA-IgA, EBNA1/IgA and combination. The antibodies VCA-IgA (Euroimmun, Lubeck, Germany) and EBNA1-IgA (Zhongshan Bio-tech, Zhongshan, China) were tested by ELISA on a TECAN Freedom EVOlyzer 200/8 platform according to the manufacturer’s specifications. EBNA1s in Zhongshan Bio-tech kit were produced with purified recombinant peptide specified by EBV BKRF1 (72 kD) (He et al., 2018). The EBV VCAs in Euroimmun kit were obtained from the pyrolysis products of human B lymphocytes (P3HR1cell line) infected by EBV (Gao et al., 2017). The levels of the antibodies were assessed by the relative optical density (rOD) calculated according to the manufacturers’ instructions by dividing the optical density (OD) value by a reference control (Ji et al., 2014). In this study, the multiplication model based on VCA-IgA and EBNA1-IgA was calculated by using the following formula: *ThelevelofcombinationVCA*-*IgA* × *EBNA*-*IgA*. The formula for PROB calculated by logistic regression based on VCA-IgA and EBNA1-IgA was as follows: *LogitPROB* =  − 3.934 + 2.203 × *VCA* − *IgA* × *EBNA* − *IgA* (Gao et al., 2017; Yu et al., 2018). In the formulas, VCA-IgA and EBNA1-IgA represent the rOD values for VCA-IgA and EBNA1-IgA, respectively, which were tested by ELISA.

The written informed consent was obtained from healthy controls. The serum samples of NPC patients were collected after clinical use which were exempted from informed consent. This study was approved by the Clinical Research Ethics Committee of the Jiangmen Central Hospital (2019–28).

### Statistical analysis

Categorical variables are described as numbers and percentages. Continuous variables are shown as the means and standard deviations (SDs). The levels of VCA-IgA, EBNA1-IgA, PROB and combination were compared by *t* tests in different population. Receiver operating characteristic (ROC) curve analysis was performed. The cut-off value of each marker was defined with the largest Youden Index selected from each ROC curve. The effects of the screening markers were measured using the sensitivity, specificity and area under the ROC curve (AUC). The base information of different populations was described and compared by the *χ*^2^ test and Fisher’s exact test. The difference in sensitivities of markers were compared by *χ*^2^ test, Fisher’s exact test and McNemar test.

The differences in AUCs were compared using the Z test according to the DeLong method (DeLong, DeLong & Clarke-Pearson, 1988). The 95% confidence intervals (CIs) of the sensitivities, specificities and AUCs were calculated. The statistical analyses were carried out using MedCalc Statistical Software version 15.2.2 (MedCalc Software bvba, Ostend, Belgium) and GraphPad Prism software version 8.0 (San Diego, CA, USA) and were two-sided, with significance set at *p* < 0.05.

**Table 1:** Characteristics of the total population.

Categories	NPC cases (*N* = 554), *n* (%)				Controls (*N* = 10,340)	
	Early-stage NPC cases (*n* = 73)	Advanced-stage NPC cases (*n* = 481)	Total	*P*^a^	*n* (%)	*P*^b^
Sex				0.356		<0.001
Male	49(67.12)	348(72.35)	397(71.66)		3,959(38.29)	
Female	24(32.88)	133(27.65)	157(28.34)		6,381(61.71)	
Age (years)				0.546		<0.001
30∼	7(9.60)	26(5.41)	33(5.96)		1,405(13.59)	
35∼	4(5.48)	35(7.28)	39(7.04)		1,439(13.92)	
40∼	6(8.22)	64(13.31)	70(12.64)		1,366(13.21)	
45∼	15(20.55)	94(19.54)	109(19.68)		1,512(14.62)	
50∼	14(19.18)	86(17.88)	100(18.05)		1,244(12.03)	
55∼	8(3.70)	73(15.18)	81(14.62)		997(9.64)	
60∼	11(15.07)	71(14.76)	82(14.80)		878(8.49)	
65∼69	8(10.96)	32(6.65)	40(7.22)		1,499(14.50)	
Smoking history				0.584		<0.001
Yes	24(32.88)	174(36.17)	198(35.74)		1,670(16.15)	
No	49(67.12)	307(63.83)	356(64.26)		8,670(83.85)	
NPC family history				0.566		<0.001
Yes	6(8.22)	50(10.4)	56(10.11)		198(1.91)	
No	67(91.78)	431(89.60)	498(89.89)		10,142(98.09)	

**Notes.**

a Differences in sex, age, smoking history and NPC family history between early-stage and advanced-stage NPC cases were compared by the *χ*^2^ test.

b Differences in the baseline information distributions of the NPC cases and controls were compared by the *χ*^2^ test.

## Results

### Baseline information

The characteristics of the 554 cases and 10340 healthy controls are shown in Table 1. In total, 554 NPC patients were enrolled in this study. Of them, 397 (71.66%) were men, and the mean age was 50.86 ±  9.48 years. Among the 554 patients, 198 (35.74%) had a smoking history, and 56 (10.11%) had a family history of NPC. Of the 10340 healthy controls, 3959 (38.29%) were men, and the mean age was 48.57 ±  11.60 years. Among the 10,340 healthy controls, 1,670 (16.15%) had a smoking history, and 198 (1.91%) had a family history of NPC (Table 1).

The differences in age, sex, smoking history and NPC family history were significant between NPC cases and healthy controls (Table 1). There were no statistically significant differences in age, sex, smoking history and NPC family history between the early-stage and advanced-stage cases (Table 1).

The characteristics of the 320 cases and 320 healthy controls in the training stage are shown in Table 2. In this stage, the controls and NPC cases were matched by sex and age to prevent bias. Differences in age, smoking history and sex were not statistically significant, while differences in NPC family history were significant between the cases and controls. There were no statistically significant differences in sex, age, smoking history, or NPC family history between the early-stage and advanced-stage cases (Table 2).

**Table 2:** Characteristics of the training stage population.

Categories		NPC cases (*N* = 320), *n* (%)					Controls (*N* = 320)	
		Early- stage NPC cases (*n* = 40)	Advanced- stage NPC cases(*n* = 280)	Total	*P*^a^		*n* (%)	*P*^b^
Sex					0.377			0.661
Male		26 (67.74)	201 (74.89)	227(70.94)			232 (72.50)	
Female		14 (32.26)	79 (25.11)	93 (29.06)			88(27.50)	
Age (years)					0.419			0.967
30∼		3 (7.50)	16 (5.71)	19 (5.94)			23 (7.19)	
35∼		2 (5.00)	23 (8.21)	25 (7.81)			30 (9.38)	
40∼		1 (2.50)	41 (14.64)	42 (13.13)			40 (12.50)	
45∼		8 (20.00)	55 (19.64)	63 (19.69)			58 (18.13)	
50∼		7 (17.50)	46 (16.43)	53 (16.56)			48 (15.00)	
55∼		6 (15.00)	42 (15.00)	48 (15.00)			45 (14.06)	
60∼		9 (22.50)	42 (15.00)	51 (15.94)			53 (16.56)	
65∼69		4 (10.00)	15 (5.36)	19 (5.94)			23 (7.19)	
Smoking history					0.824			0.235
Yes		13 (32.50)	96 (34.29)	109 (34.06)			95 (29.69)	
No		27 (67.50)	184 (65.71)	211 (65.94)			225 (70.31)	
NPC family history					0.097			<0.001
Yes		1 (2.50)	32 (11.42)	33 (10.31)			6 (1.88)	
No		39 (97.50)	248(88.58)	287 (89.69)			314 (98.12)	

**Notes.**

a Differences in sex and smoking history between early-stage and advanced-stage NPC cases were compared by the *χ*^2^ test. Differences in age and NPC family history between early-stage and advanced-stage NPC cases were compared by Fisher’s exact test.

b Differences in the baseline information distributions of the NPC cases and controls were compared by the *χ*^2^ test.

**Figure 1:**
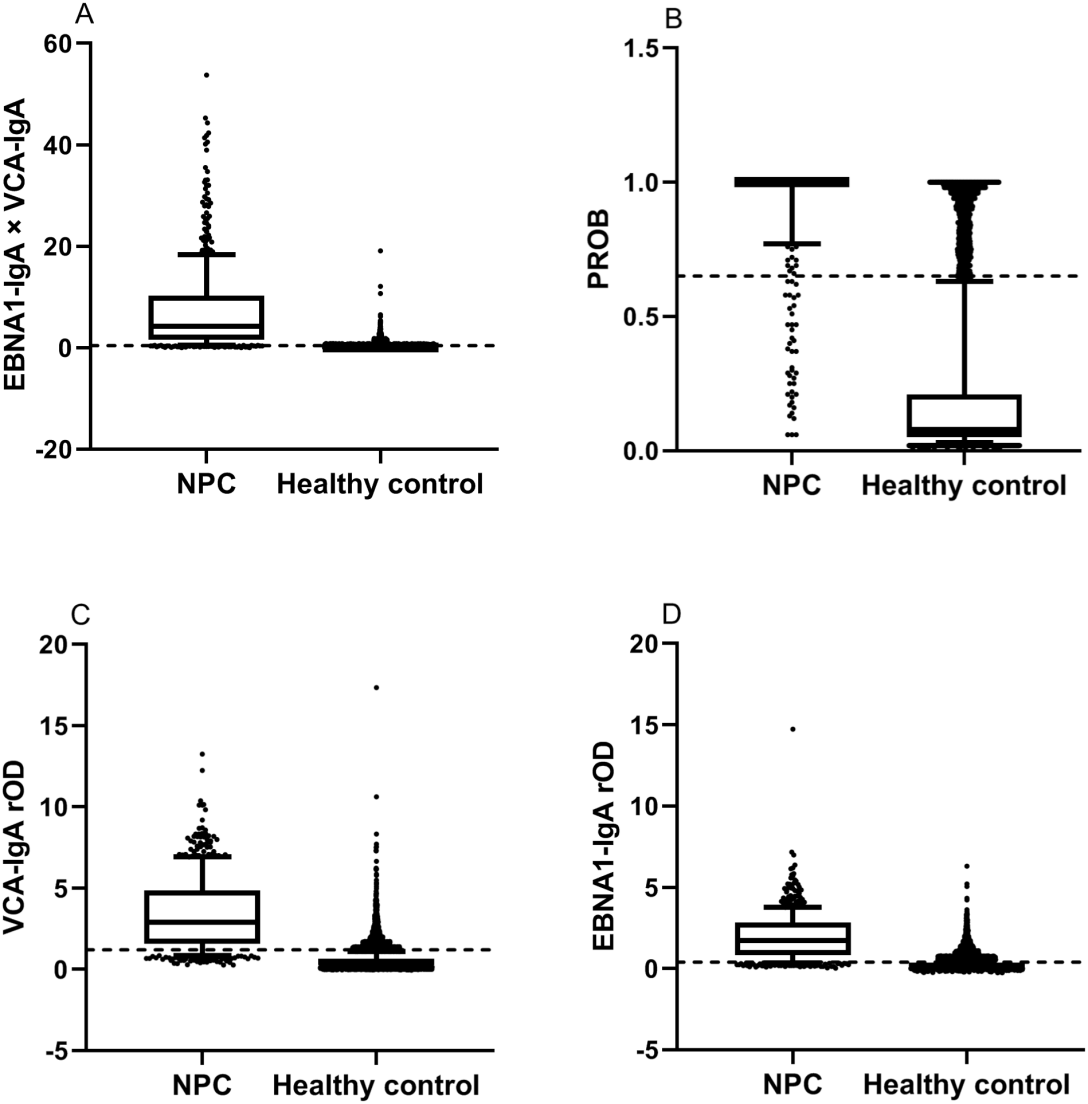
Comparison of levels of markers in NPC patients and healthy controls by *t* tests. The dotted lines represent cut-off values of the markers. Each box indicates 25/75 percentiles. Whisker caps represent 10/90 percentiles. (A) The level of the combination. The level of the combination for NPC patients was higher than for healthy controls by the t test (p<0.001). (B) The level of the PROB. The level of the PROB for NPC patients was higher than for healthy controls by the t test (p<0.001). (C) The level of the VCA-IgA. The level of the VCA-IgA for NPC patients was higher than for healthy controls by the t test (p<0.001). (D) The level of the EBNA1-IgA. The level of the EBNA1-IgA for NPC patients was higher than for healthy controls by the t test (p<0.001).

In the verification stage, a total of 10,254 subjects were enrolled, including 234 NPC cases and 10,020 healthy controls. Of the 10,254 subjects, 3,897 (38.00%) were men, and the mean age was 48.57 ±  11.61 years. Among the 10,254 subjects, 1,664 (16.23%) had a smoking history, and 215 (2.10%) had a family history of NPC.

**Figure 2:**
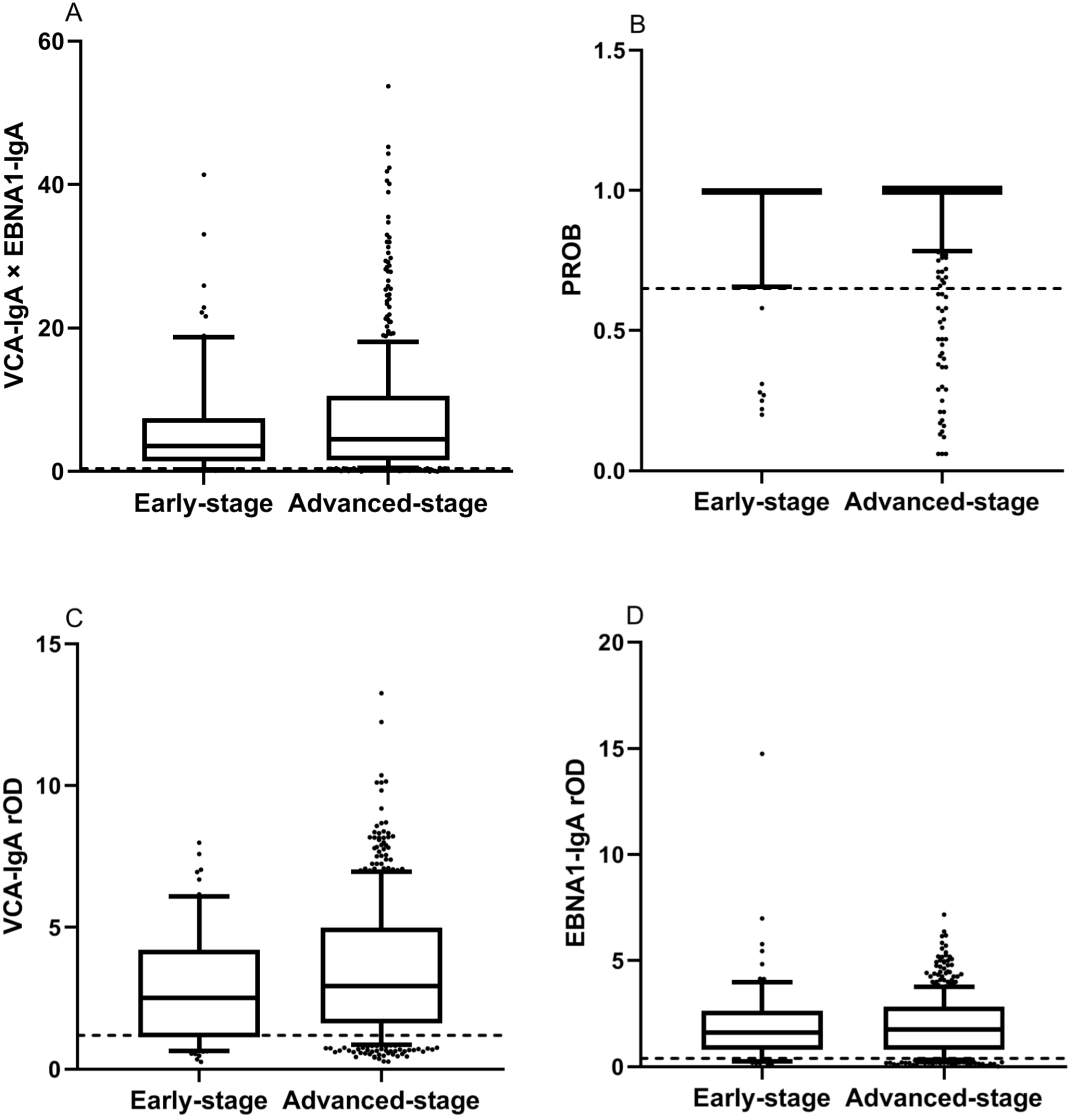
Comparison of levels of markers in early-stage and advanced-stage NPC patients by *t* tests. The dotted lines represent cut-off values of the markers. Each box indicates 25/75 percentiles. Whisker caps represent 10/90 percentiles. (A) The level of the combination. The difference in combination was not significant between early-stage and advanced-stage NPC patients by the t test (p>0.05). (B) The level of the PROB. The difference in PROB was not significant between early-stage and advanced-stage NPC patients by the t test (p>0.05). (C) The level of the VCA-IgA. The difference in VCA-IgA was not significant between early-stage and advanced-stage NPC patients by the t test (p>0.05). (D) The level of the EBNA1-IgA. The difference in EBNA1-IgA was not significant between early-stage and advanced-stage NPC patients by the t test (p>0.05).

### Comparison of levels of markers in NPC patients and healthy controls

The rODs of VCA-IgA and EBNA1-IgA, PROB value and combination value in NPC patients and healthy controls were showed in Fig. 1. The *t* tests showed that the means of markers in NPC patients were all significantly higher than those in healthy controls (*p* < 0.001).

### Comparison of levels of markers in early-stage and advanced-stage NPC patients

Of the 554 NPC patients, 73 (13.18%) were early-stage. The levels of VCA-IgA EBNA1-IgA, PROB and combination in early-stage and advanced-stage NPC patients were showed in Fig. 2. The differences in VCA-IgA, EBNA1-IgA, PROB and combination were not significant between early-stage and advanced-stage NPC patients by *t* tests (*p* > 0.05).

### Diagnostic value of the markers

The diagnostic performance of the markers is shown in Table 3 by using training samples. The combination achieved a sensitivity of 90.94% (95% CI [87.2%–93.8%]), a specificity of 92.50% (95% CI [89.0%–95.1%]) and an AUC of 0.978 (95% CI [0.969– 0.986]). The PROB achieved a sensitivity of 87.81% (95% CI [83.7%–91.2%]), a specificity of 95.94% (95% CI [93.2%–97.8%]) and an AUC of 0.972 (95% CI [0.962– 0.982]). The VCA-IgA had a sensitivity of 84.06% (95% CI [79.6%–87.9%]), a specificity of 91.25% (95% CI [87.6%–94.1%]) and an AUC of 0.947 (95% CI [0.932– 0.963]). The sensitivity, specificity and AUC of EBNA1-IgA were 87.81% (95% CI [83.7%–91.2%]), 85.00% (95% CI [80.6%–88.7%]), and 0.935 (95% CI [0.917–0.953]), respectively.

**Figure 3:**
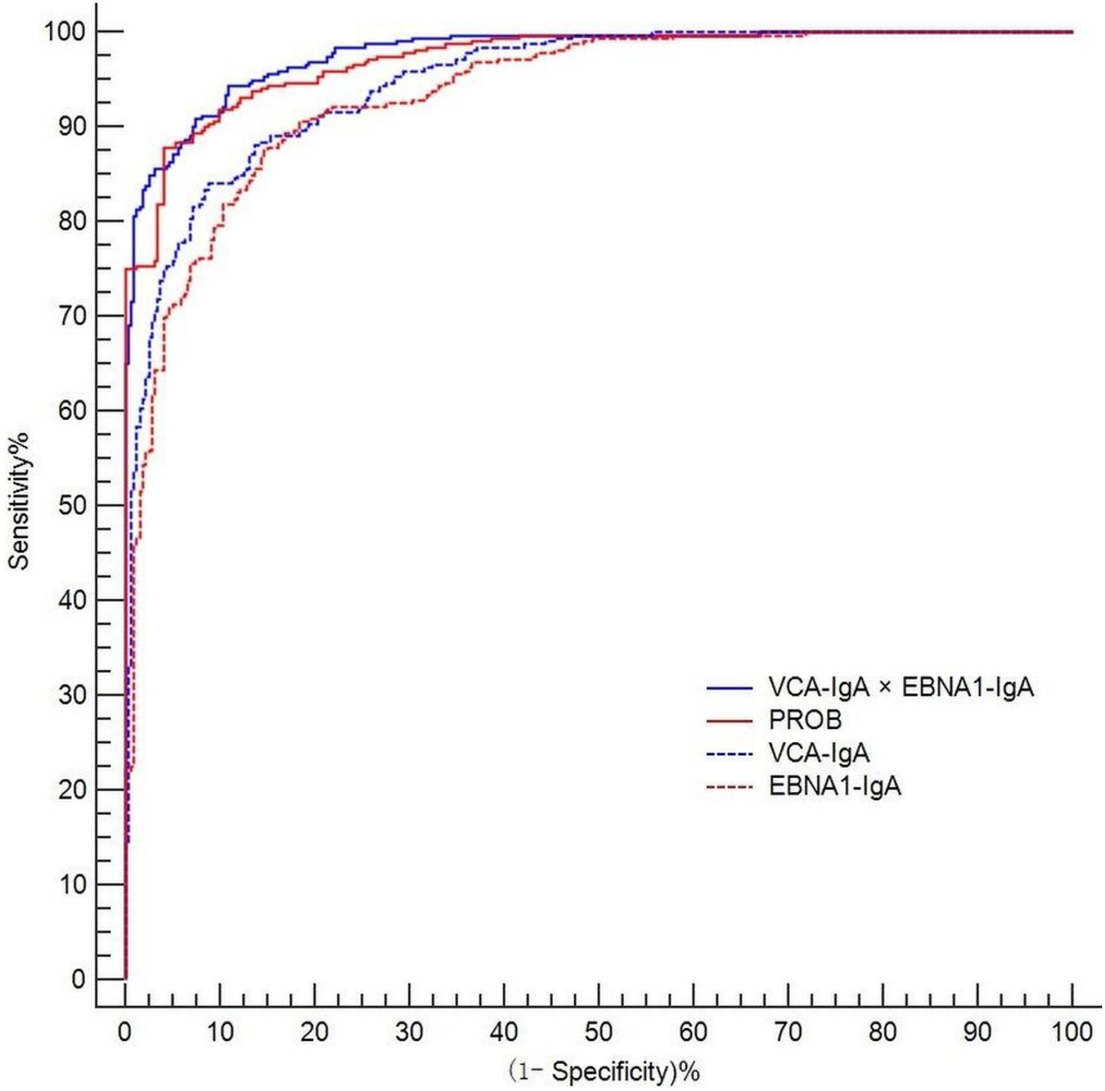
ROC curves for combination, PROB, VCA-IgA and EBNA1-IgA. The axes are expressed as percentages.

Compared to the AUCs of VCA-IgA (*p* < 0.001) ,EBNA1-IgA ( *p* < 0.001), and PROB (*p* < 0.01), the combination yielded a higher AUC (Table 3 and Fig. 3) by using training samples. The differences in the sensitivities of the markers between early-stage and advanced-stage NPC patients were not significant by using verification samples (*p* > 0.05, Table 4). Compared with each marker alone by McNemar test, the combination had a higher sensitivity for early-stage NPC patients (Table 4).

The differences in sensitivities of EBNA1-IgA, PROB and the combination between man and female NPC patients were not significant by using verification samples (*p* > 0.05), while the sensitivity of VCA-IgA in man NPC patients was higher than in female NPC patients (*p* = 0.047, Table 5). The sensitivity differences of the markers in different age, smoking history and NPC family history were not statistically significant by by using verification samples (*p* > 0.05, Tables 6–8).

**Table 3:** Diagnostic value of the markers.

Marker	Cut-off value	Sensitivity (%) (95% CI)	Specificity (%) (95% CI)	AUC (95% CI)	P^a^
VCA-IgA × EBNA1-IgA	0.429	90.94 (87.2, 93.8)	92.50 (89.0, 95.1)	0.978 (0.969, 0.986)	
PROB	0.949	87.81 (83.7, 91.2)	95.94 (93.2, 97.8)	0.972 (0.962, 0.982)	<0.01
VCA-IgA	1.194	84.06 (79.6, 87.9)	91.25 (87.6, 94.1)	0.947 (0.932, 0.963)	<0.001
EBNA1-IgA	0.397	87.81 (83.7, 91.2)	85.00(80.6, 88.7)	0.935(0.917, 0.953)	<0.001

**Notes.**

a The AUC comparisons of the markers were performed by the *Z* test.

**Table 4:** Sensitivity differences for early-stage and advanced-stage NPC by using different markers.

Marker	Cut-off value	Sensitivity (%)		*P*^a^
		Early-stage NPC cases	Advanced- stage NPC cases	
VCA-IgA × EBNA1-IgA	0.429	93.94	91.04	0.747
PROB	0.949	84.85^b^	84.08	1.000
VCA-IgA	1.194	75.76^c^	82.59	0.339
EBNA1-IgA	0.397	84.85^d^	86.07	0.791

**Notes.**

a The sensitivity differences between early-stage and advanced-stage NPC were compared by Fisher’s exact test

b Compared with combination, the PROB had a lower sensitivity for early-stage NPC patients by McNemar test (*p* < 0.001).

c Compared with combination, the VCA-IgA had a lower sensitivity for early-stage NPC patients by McNemar test (*p* < 0.001).

d Compared with combination, the EBNA1-IgA had a lower sensitivity for early-stage NPC patients by McNemar test (*p* < 0.001).

### Verifying the effect of the combination on NPC screening

A total of 10,253 subjects were enrolled to verify the NPC screening effect, including 234 NPC cases and 10,020 healthy controls sourced from the screening field. In this stage, the combination achieved an overall sensitivity of 91.45% (214/234), a sensitivity for early-stage NPC detection of 93.94% (31/33), a specificity of 93.45% (9364/10020), and an AUC of 0.978 (95% CI [0.968–0.987]).

**Table 5:** Sensitivity differences for man and female NPC by using different markers.

Marker	Cut-off value	Sensitivity (%) for NPC cases		*P*^a^
		Man	Female	
VCA-IgA^*x*^ EBNA1-IgA	0.429	91.76	90.63	0.781
PROB	0.949	85.29	81.25	0.450
VCA-IgA	1.194	84.71	73.44	0.047
EBNA1-IgA	0.397	85.88	85.93	0.991

**Notes.**

a The sensitivity differences between man and female NPC were compared by *χ*^2^ test.

**Table 6:** Sensitivity differences for different ages of NPC patients by using different markers.

Marker	Sensitivity (%) for different ages (years) of NPC patients								*P*^a^
	30∼	35∼	40∼	45∼	50∼	55∼	60∼	65∼69	
VCA-IgA × EBNA1-IgA	100.00	85.71	89.29	91.30	85.11	100.00	93.55	90.48	0.274
PROB	85.71	78.57	78.57	84.78	83.00	90.91	87.10	80.95	0.904
VCA-IgA	78.57	78.57	85.71	80.43	78.72	84.85	87.10	76.19	0.950
EBNA1-IgA	92.86	78.57	85.71	86.96	85.11	93.94	83.87	76.19	0.674

**Notes.**

a The sensitivity differences in different ages of NPC patients were compared by Fisher’s exact test.

**Table 7:** Sensitivity differences for different smoking history NPC by using different markers.

Marker	Cut-off value	Sensitivity (%) for NPC		*P*^a^
		smoking	no smoking	
VCA-IgA^*x*^ EBNA1-IgA	0.429	91.01	91.72	0.850
PROB	0.949	87.64	82.07	0.257
VCA-IgA	1.194	85.39	79.31	0.243
EBNA1-IgA	0.397	86.52	85.52	0.831

**Notes.**

a The sensitivity differences for different smoking history NPC were compared by *χ*^2^ test.

**Table 8:** Sensitivity differences for NPC with and without NPC family history by using different markers.

Marker	Cut-off value	Sensitivity (%)		*P*^a^
		NPC with NPC family history	NPC without NPC family history	
VCA-IgA^×^				
EBNA1-IgA	0.429	91.30	91.47	1.000
PROB	0.949	78.26	84.83	0.412
VCA-IgA	1.194	86.96	81.04	0.487
EBNA1-IgA	0.397	73.91	87.02	0.082

**Notes.**

a The sensitivity differences between NPC with and without NPC family history were compared by *χ*^2^ test.

## Discussion

NPC is a main health problem that leads to a high health burden in southern China, especially in Guangdong province (Cao, Simons & Qian, 2011). EBV antibodies are widely applied in NPC screening. PROB calculated by logistic model were applied in NPC screening based on VCA-IgA and EBNA1-IgA (Gao et al., 2017; Liu et al., 2013; Yu et al., 2018). In this study, a combination calculated by multiplication model based on VCA-IgA and EBNA1-IgA was applied to NPC screening. The NPC screening effect of combination was evaluated and compared with the individual screening markers, PROB, VCA-IgA and EBNA1-IgA. Compared with PROB, VCA-IgA and EBNA1-IgA, the combination had a higher AUC of 0.978 (95% CI [0.969–0.986]). We found that the combination calculated by using a multiplication model can be applied to NPC screening.

In this study, a large number of healthy controls and 554 NPC patients were collected from the endemic areas, which is favourable for evaluating the performance of the markers for NPC screening. In the verification stage, 10,254 subjects were enrolled to verify the NPC screening effect. The combination achieved a sensitivity of 91.45%, a specificity of 93.45% and an AUC of 0.978 (95% CI [0.968–0.987]). These results demonstrated that the combination calculated by using a multiplication model was effective for NPC screening.

In this study, the levels of markers (PROB, VCA-IgA, EBNA1-IgA and combination) in NPC patients were higher than in healthy controls. It was consistent with the results of previous reports (Liu et al., 2012). We found the difference in sensitivities of the combination in different age, sex, smoking history and NPC family history were not statistically significant. The VCA-IgA had a higher sensitivity for man NPC patients than female NPC patients by using verification samples. Since the *P* value (0.047) was very close to 0.05, and the verification sample size was not very large. The difference in sensitivity of VCA-IgA between man and female NPC patients may be due to the random fluctuation.

In the present study, the AUCS, sensitivities and specificities of VCA-IgA and EBNA1-IgA were lower than those of the combination, showing that the combination was more effective in diagnosis. The AUC of the combination was a little bit larger than the PROB. There was a slight increase (3.13%) in the sensitivity of the combination compared to the sensitivity of the PROB. The specificity was lower for the combination (92.50%) than for the PROB (95.94%). In areas with high NPC incidence, the increased sensitivity means that more early-stage NPC patients will be detected and treated early, while the decreased specificity may lead to an increased false positive rate and increased costs of the screening program.

The present study had some limitations. First, there was some bias in identifying the 10340 subjects as healthy controls because not all healthy controls underwent an examination by fibreoptic endoscopic examination. Second, since the study population was obtained from provinces with a high risk of NPC, the results may be limited for application in other populations. Third, the sample size of the early-stage NPC patients was not large enough in this study. There was some bias in estimating sensitivity for early-stage NPC patients.

## Conclusions

We successfully developed a combination of two ELISA tests based on VCA-IgA and EBNA1-IgA to improve the effect of NPC screening by using a multiplication model. Compared with VCA-IgA and EBNA1-IgA individually, the combination had an improved diagnostic performance. The AUC and sensitivity of the combination were slightly higher than those of the PROB, while the specificity was lower for the combination than for the PROB. The results suggested that the combination was effective and can be an option for NPC screening.

##  Supplemental Information

10.7717/peerj.10254/supp-1 Supplemental Information 1 Raw data

## Additional Information and Declarations

### Data Availability

The following information was supplied regarding data availability: The raw measurements are available in the Supplementary Files.

## References

[ref-1] Cao SM, Simons MJ, Qian CN (2011). The prevalence and prevention of nasopharyngeal carcinoma in China. Chinese Journal of Cancer.

[ref-2] Chang ET, Adami HO (2006). The enigmatic epidemiology of nasopharyngeal carcinoma. Cancer Epidemiol Biomarkers Prev.

[ref-3] Chang ET, Liu Z, Hildesheim A, Liu Q, Cai Y, Zhang Z, Chen G, Xie SH, Cao SM, Shao JY, Jia WH, Zheng Y, Liao J, Chen Y, Lin L, Ernberg I, Vaughan TL, Adami HO, Huang G, Zeng Y, Zeng YX, Ye W (2017). Active and passive smoking and risk of nasopharyngeal carcinoma: a population-based case-control study in Southern China. American Journal of Epidemiology.

[ref-4] Chen YP, Chan ATC, Le QT, Blanchard P, Sun Y, Ma J (2019). Nasopharyngeal carcinoma. Lancet.

[ref-5] Chien YC, Chen JY, Liu MY, Yang HI, Hsu MM, Chen CJ, Yang CS (2001). Serologic markers of Epstein-Barr virus infection and nasopharyngeal carcinoma in Taiwanese men. New England Journal of Medicine.

[ref-6] Choi CW, Lee MC, Ng WT, Law LY, Yau TK, Lee AW (2011). An analysis of the efficacy of serial screening for familial nasopharyngeal carcinoma based on Markov chain models. Familial Cancer.

[ref-7] DeLong ER, DeLong DM, Clarke-Pearson DL (1988). Comparing the areas under two or more correlated receiver operating characteristic curves: a nonparametric approach. Biometrics.

[ref-8] Enyedi A, Csongrádi A, Altorjay IT, Beke GL, Váradi C, Enyedi EE, Kiss DR, Bányai E, Kalina E, Kappelmayer J, Tóth A, Papp Z, Takács I, Fagyas M (2020). Combined application of angiotensin converting enzyme and chitotriosidase analysis improves the laboratory diagnosis of sarcoidosis. Clinica Chimica Acta.

[ref-9] Fachiroh J, Schouten T, Hariwiyanto B, Paramita DK, Harijadi A, Haryana SM, Ng MH, Middeldorp JM (2004). Molecular diversity of Epstein-Barr virus IgG and IgA antibody responses in nasopharyngeal carcinoma: a comparison of Indonesian, Chinese, and European subjects. Journal of Infectious Diseases.

[ref-10] Gao R, Wang L, Liu Q, Zhang LF, Ye YF, Xie SH, Du JL, Chen SH, Guo J, Yang MJ, Lin CY, Cao SM (2017). Evaluation of seven recombinant VCA-IgA ELISA kits for the diagnosis of nasopharyngeal carcinoma in China: a case-control trial. BMJ Open.

[ref-11] Gulley ML (2001). Molecular diagnosis of Epstein-Barr virus-related diseases. Journal of Molecular Diagnostics.

[ref-12] He YQ, Xue WQ, Xu FH, Xu YF, Zhang JB, Yu HL, Feng QS, Chen LZ, Cao SM, Liu Q, Mu J, Zeng YX, Jia WH (2018). The relationship between environmental factors and the profile of epstein-barr virus antibodies in the lytic and latent infection periods in healthy populations from endemic and non-endemic nasopharyngeal carcinoma areas in China. EBioMedicine.

[ref-13] Henle G, Henle W (1976). Epstein-Barr virus-specific IgA serum antibodies as an outstanding feature of nasopharyngeal carcinoma. International Journal of Cancer.

[ref-14] Ji MF, Huang QH, Yu X, Liu Z, Li X, Zhang LF, Wang P, Xie SH, Rao HL, Fang F, Guo X, Liu Q, Hong MH, Ye W, Zeng YX, Cao SM (2014). Evaluation of plasma Epstein-Barr virus DNA load to distinguish nasopharyngeal carcinoma patients from healthy high-risk populations in Southern China. Cancer.

[ref-15] Ji MF, Sheng W, Cheng WM, Ng MH, Wu BH, Yu X, Wei KR, Li FG, Lian SF, Wang PP, Quan W, Deng L, Li XH, Liu XD, Xie YL, Huang SJ, Ge SX, Huang SL, Liang XJ, He SM, Huang HW, Xia SL, Ng PS, Chen HL, Xie SH, Liu Q, Hong MH, Ma J, Yuan Y, Xia NS, Zhang J, Cao SM (2019). Incidence and mortality of nasopharyngeal carcinoma: interim analysis of a cluster randomized controlled screening trial (PRO-NPC-001) in southern China. Annals of Oncology.

[ref-16] Ji MF, Wang DK, Yu YL, Guo YQ, Liang JS, Cheng WM, Zong YS, Chan KH, Ng SP, Wei WI, Chua DT, Sham JS, Ng MH (2007). Sustained elevation of Epstein-Barr virus antibody levels preceding clinical onset of nasopharyngeal carcinoma. British Journal of Cancer.

[ref-17] Jia WH, Huang QH, Liao J, Ye W, Shugart YY, Liu Q, Chen LZ, Li YH, Lin X, Wen FL, Adami HO, Zeng Y, Zeng YX (2006). Trends in incidence and mortality of nasopharyngeal carcinoma over a 20-25 year period (1978/1983-2002) in Sihui and Cangwu counties in southern China. BMC Cancer.

[ref-18] Lin ZX, Yang ZN, Zhan YZ, Xie WJ, Li GW, Feng HT (2009). Application study of the 2008 staging system of nasopharyngeal carcinoma. Ai Zheng.

[ref-19] Liu Y, Huang Q, Liu W, Liu Q, Jia W, Chang E, Chen F, Liu Z, Guo X, Mo H, Chen J, Rao D, Ye W, Cao S, Hong M (2012). Establishment of VCA and EBNA1 IgA-based combination by enzyme-linked immunosorbent assay as preferred screening method for nasopharyngeal carcinoma: a two-stage design with a preliminary performance study and a mass screening in southern China. International Journal of Cancer.

[ref-20] Liu Z, Ji MF, Huang QH, Fang F, Liu Q, Jia WH, Guo X, Xie SH, Chen F, Liu Y, Mo HY, Liu WL, Yu YL, Cheng WM, Yang YY, Wu BH, Wei KR, Ling W, Lin X, Lin EH, Ye W, Hong MH, Zeng YX, Cao SM (2013). Two Epstein-Barr virus-related serologic antibody tests in nasopharyngeal carcinoma screening: results from the initial phase of a cluster randomized controlled trial in Southern China. American Journal of Epidemiology.

[ref-21] Ng WT, Choi CW, Lee MC, Law LY, Yau TK, Lee AW (2010). Outcomes of nasopharyngeal carcinoma screening for high risk family members in Hong Kong. Fam Cancer.

[ref-22] Ng WT, Yau TK, Yung RW, Sze WM, Tsang AH, Law AL, Lee AW (2005). Screening for family members of patients with nasopharyngeal carcinoma. International Journal of Cancer.

[ref-23] Sam CK, Abu-Samah AJ, Prasad U (1994). IgA/VCA as a follow-up marker in the monitoring of nasopharyngeal carcinoma. European Journal of Surgical Oncology.

[ref-24] Tan LP, Tan GW, Sivanesan VM, Goh SL, Ng XJ, Lim CS, Kim WR, Mohidin T, Mohd Dali NS, Ong SH, Wong CY, Sawali H, Yap YY, Hassan F, Pua KC, Koay CE, Ng CC, Khoo AS, Malaysian Nasopharyngeal Carcinoma Study Group (2020). Systematic comparison of plasma EBV DNA, anti-EBV antibodies and miRNA levels for early detection and prognosis of nasopharyngeal carcinoma. International Journal of Cancer.

[ref-26] Wei KR, Zheng RS, Zhang SW, Liang ZH, Li ZM, Chen WQ (2017). Nasopharyngeal carcinoma incidence and mortality in China, 2013. Chinese Journal of Cancer.

[ref-25] Torre LA, Bray F, Siegel RL, Ferlay J, Lortet-Tieulent J, Jemal A (2015). Global cancer statistics, 2012. CA: a Cancer Journal for Clinicians.

[ref-27] Wei WI, Sham JST (2005). Nasopharyngeal carcinoma. The Lancet.

[ref-28] Yang XR, Diehl S, Pfeiffer R, Chen CJ, Hsu WL, Dosemeci M, Cheng YJ, Sun B, Goldstein AM, Hildesheim A, Chinese and American Genetic Epidemiology of NPC Study Team (2005). Evaluation of risk factors for nasopharyngeal carcinoma in high-risk nasopharyngeal carcinoma families in Taiwan. Cancer Epidemiology, Biomarkers & Prevention.

[ref-29] Yong SK, Ha TC, Yeo MC, Gaborieau V, McKay JD, Wee J (2017). Associations of lifestyle and diet with the risk of nasopharyngeal carcinoma in Singapore: a case-control study. Chinese Journal of Cancer.

[ref-31] Yu X, Ji M, Cheng W, Wu B, Du Y, Cao S (2018). Assessment of the long-term diagnostic performance of a new serological screening scheme in large-scale nasopharyngeal carcinoma screening. Journal of Cancer.

[ref-30] Yu MC, Yuan JM (2002). Epidemiology of nasopharyngeal carcinoma. Seminars in Cancer Biology.

[ref-32] Zeng Y, Zhang LG, Li HY, Jan MG, Zhang Q, Wu YC, Wang YS, Su GR (1982). Serological mass survey for early detection of nasopharyngeal carcinoma in Wuzhou City, China. International Journal of Cancer.

